# Comparison and evaluation of overcoring and hydraulic fracturing stress measurements

**DOI:** 10.1038/s41598-024-59550-1

**Published:** 2024-04-16

**Authors:** Peng Li, Meifeng Cai, Shengjun Miao, Yuan Li, Liang Sun, Jiangtao Wang, Mostafa Gorjian

**Affiliations:** 1 https://ror.org/02egmk993 grid.69775.3a 0000 0004 0369 0705 Key Laboratory of Ministry of Education for Efficient Mining and Safety of Metal Mines, University of Science and Technology Beijing, No.30 Xueyuan Road, Haidian District, Beijing, 100083 China; 2 grid.411510.0 0000 0000 9030 231X State Key Laboratory of Coal Resources and Safe Mining, China University of Mining and Technology, Xuzhou, 221116 China; 3 https://ror.org/02egmk993 grid.69775.3a 0000 0004 0369 0705 Key Laboratory of Intelligent Bionic Unmanned Systems of Ministry of Education, University of Science and Technology Beijing, No.30 Xueyuan Road, Haidian District, Beijing, 100083 China; 4 https://ror.org/03rmrcq20 grid.17091.3e 0000 0001 2288 9830 .Geological Engineering, Department of Earth, Ocean and Atmospheric Sciences, University of British Columbia, Vancouver, BC Canada

**Keywords:** Overcoring, Hydraulic fracturing, Stress measurements, Improved Bayesian regression approach, Kolmogorov–Smirnov test, Civil engineering, Statistics

## Abstract

The stress measurements determined by both the overcoring (OC) and hydraulic fracturing (HF) methods in the Shuichang iron mine and Sanshandao gold mine were compared and evaluated, respectively. The results indicate that the independent OC and HF data in the two mines reveal the same dominant faulting stress regime. The *σ*_H_ orientations derived from the OC and HF methods in the Shuichang iron mine are dominantly oriented in the N81.1°W–N89.4°W and N77.0°E–N88.0°E, respectively, and the *σ*_H_ orientations yielded from the OC and HF techniques in the Sanshandao gold mine are predominantly in the N30°W–N90°W and N55.5°W–N60.4°W, respectively; hence, the *σ*_H_ orientations obtained by the two different methods in the two mines are comparatively similar. In addition, the shapes of the probability density diagrams using an improved Bayesian regression approach of the three principal stresses measured by the OC and HF methods in the same mine are quite similar, and all the obtained Kolmogorov–Smirnov test *p*-values are larger than the selected significance level of 0.01, indicating that the stress data interpreted by the two methods approximately follow the same distribution law. Thus, the performance of the two techniques and the reliability of the measured data are satisfactory.

## Introduction

In-situ stress is a natural original stress that existed in the stratum before engineering excavation. The measurement of in-situ stress and its distribution characteristics are the major concerns of rock mechanics and rock engineering, which involves the understanding of basic geological processes, such as plate tectonics and earthquakes, as well as the engineering structural design in and on rock masses^1–5^. Numerous theoretical studies and engineering practices indicate that in-situ stress is the fundamental force that causes deformation and failure of various rock engineering, and it is an important basis for determining the mechanical properties of engineering rock masses, analyzing the stability of surrounding rocks, and optimizing engineering design, construction, and operation, and particularly the rock mechanics analysis of engineering problems requires stress tensor itself as a boundary condition^6^. Moreover, the evolution of crustal geological structure and various physical and chemical phenomena in the earth are closely related to in-situ stress, which directly affects and even dominates crustal movement and geological processes^7–10^. This means that the in-situ stress state should be considered in the overall geological and engineering environment. As a result, there is increasing awareness of the significance of the in-situ stress in rock engineering applications and seismic geological research.

It is well known that the in-situ stress state in the crust is extremely complex and changeable since the earth has experienced countless tectonic movements with different intensities and scales, and even at the same depth level in the same area, the stress state of different measuring points may be considerably distinct, so it is impossible to obtain accurate in-situ stress data through mathematical calculations or other speculative approaches. Currently, the only way to understand the stress state of a certain area is to conduct on-site in-situ stress measurement. Since the 1930s, the theories and methods of in-situ stress measurement have made significant progress worldwide. There are dozens of in-situ stress measurement and monitoring methods, especially hundreds of developed in-situ stress measurement instruments^11^. Hydraulic fracturing, overcoring, borehole breakouts, strain recovery, geological observational, and earthquake focal mechanisms methods are commonly used in in-situ stress measurement at present^12–15^, among which hydraulic fracturing and overcoring methods are the most representative mature techniques and are also recommended by ISRM^16,17^. However, in-situ stress is a difficult quantity to measure because various factors affect the origin of in-situ stress. Theoretically, stress is a property of a point, and the stress state of a point is usually characterized by the magnitudes and directions of the three principal stresses. If we want to measure the stress state and use it with any degree of confidence, the contributory factors must be understood. Due to the special nature of in-situ stress, at least two or even more methods should be adopted to estimate it in a progressive process. For example, it is suggested that the hydraulic fracturing technique should be used in the initial planning of engineering projects, and then overcoring measurements should be carried out to obtain a more refined description of the in-situ stress state. Generally, combining various direct and/or indirect methods to complement each other according to their respective attributes is helpful to obtain more reliable estimates of in-situ stresses and impose more rigorous constraints on the in-situ stress field. The benefits of using hybrid stress measurement methods have reached a consensus. However, there may be differences or even contradictions between the in-situ stress data obtained by different measurement methods, and the reliability of different types of stress data lacks rigorous, objective, and quantitative comparative analysis approaches.

In the present study, both the overcoring and hydraulic fracturing methods were employed to determine the stress state in the Shuichang iron mine and the Sanshandao gold mine, and effective stress data were identified. Notably, in-situ stress measurements using two methods in the same mine are relatively rare because of the high cost. The two kinds of stress indicators in these two mines provide us with an excellent opportunity to compare the measurement results of these two methods. Hence, in this article, the magnitude and direction of the overcoring and hydraulic fracturing stress data obtained in the two mines are compared intuitively, and then a comprehensive comparison and evaluation approach based on an improved Bayesian quantification, as well as an improved Kolmogorov–Smirnov statistical test are proposed to further quantitatively compare and analyze the overcoring and hydraulic fracturing stress measurements of these two mines, achieving good results. This is of great significance to further understand the stress scale represented by these two representative methods and improve their measurement accuracy. It should be noted that the stress data of the two mines used in this study have been employed to reveal the distribution characteristics of the in-situ stress field and its interaction mechanism with regional geological structures in these two mining areas^18,19^, which is completely different from the purpose of this study.

## In-situ stress measurements

### Overcoring

The in-situ stress measurement campaigns were conducted using an improved overcoring (OC) technique in the Shuichang iron mine and the Sanshandao gold mine. The OC technique belongs to a borehole relief method and is a relatively mature in-situ stress measurement method that has been developed for nearly 60 years. Also, it is currently the only method that can accurately determine the complete three-dimensional stress tensor of a point through a single borehole measurement, which can provide a refined description of the in-situ stress field^19,20^. The main idea behind the OC technique is to isolate the rock sample from the stress field of the surrounding rock mass in part or in whole and monitor its re-equilibrium deformation response^5,14,16^. Stress determination requires in-situ recovered strains at a circular pilot borehole wall induced by stress relief to be converted into stresses using the deformability parameters at the measurement point. Notably, the quality of measurement depends on how to solve technical problems such as drilling, gluing, and overcoring, as well as how good the rock characteristics such as anisotropy, discontinuity, and heterogeneity are known^21,22^. To date, there are many variations of overcoring measurements.

**Figure 1:**
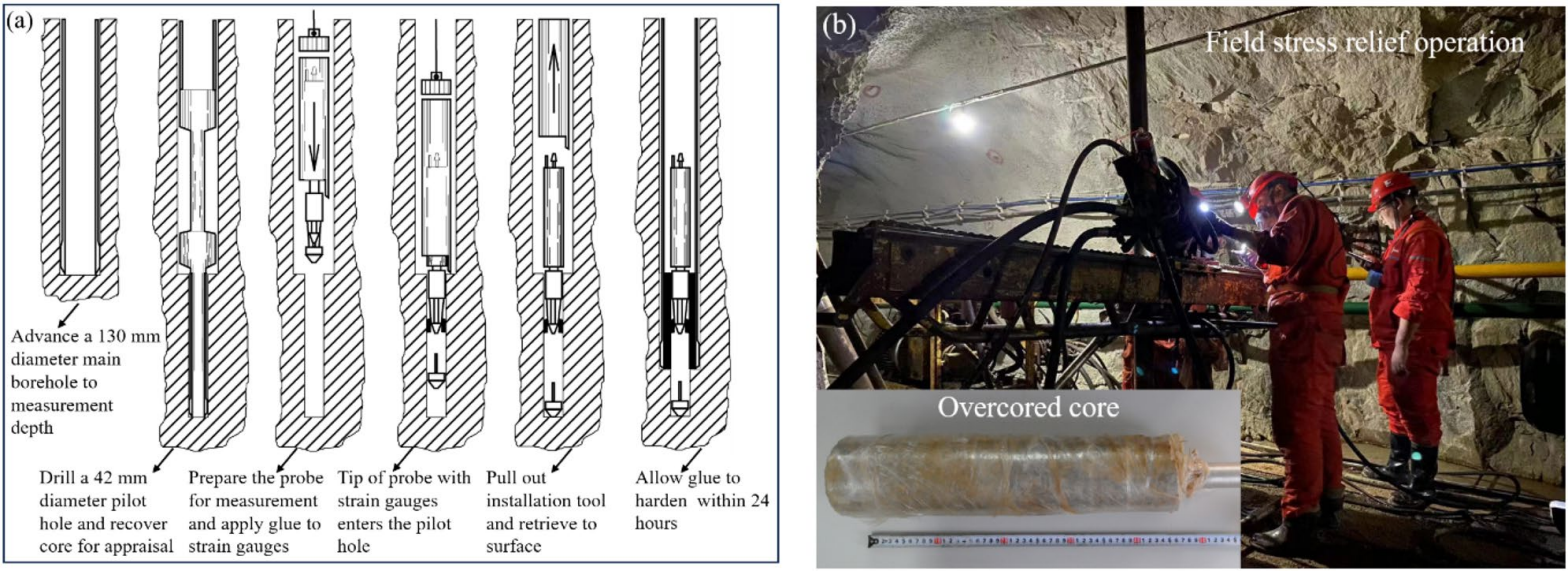
Illustrations of the measurement procedure for the OC method (**a**) (after Sjöberg et al.^16^) and the field stress relief operations (**b**).

The OC technique includes drilling a large hole first, then drilling a small-diameter pilot hole concentrically at the bottom of the large hole, and installing a hollow inclusion strain gauge at the bottom of the small hole; afterward, the hollow inclusion strain gauge is overcored using a larger coring bit, thus effectively relieving the stress acting on the hollow rock cylinder (Fig. 1). For mines in production, the application of OC method to measure in-situ rock stresses has unique advantages and is the most economical and reasonable because underground accesses are available. The prominent advantage of the hollow inclusion strain gauge involved in this method is that the strain gauge and the borehole wall are cemented together on a considerable area, resulting in good cementing quality. At the same time, the cementing agent can also be injected into the cracks and defects in the rock mass around the strain gauge to make the rock integrated, so it is easier to obtain complete overcored rocks. Hence, the strain gauge can be used in moderately fractured and soft rock masses and has good waterproof performance. As a consequence, the hollow inclusion strain gauge has become the most widely adopted in-situ stress relief measurement instrument worldwide.

However, like many other strain measuring instruments, the hollow inclusion strain gauge uses a resistance strain gauge as the sensing element, and the resistance strain gauge is very susceptive to temperature changes. The temperature changes of rocks or strain cells during overcoring have adverse effects on the measurement results, and thus it is crucial to take reasonable temperature compensation measures to eliminate the impact of temperature changes on the measurement results. Theoretical analysis and experimental results indicate that the traditional temperature compensation methods which use dummy gauges are not effective for temperature compensation of cemented strain gauges^23,24^. Against this circumstance, a new temperature compensation technique was invented, and the structure of the conventional hollow inclusion strain gauge was improved accordingly, a more detailed description of the techniques can be found in Cai et al.^25^. These new techniques have been utilized in the stress measurement activities of the two mines.

**Figure 2:**
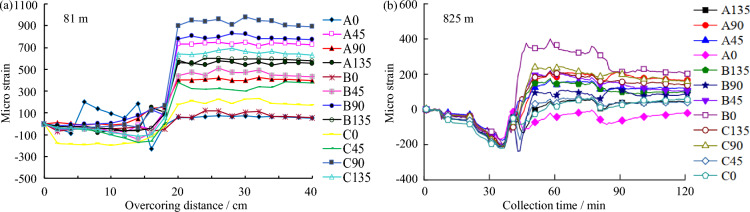
Typical stress relief curves during the OC test in the Shuichang iron mine (**a**) and the Sanshandao gold mine (**b**).

The OC equipment, setup, and measuring procedures adopted in the two mines strictly followed the suggestions of ISRM^16^. In the OC stress measurement, all measuring points were arranged within complete or basically complete rock bodies, far away from rock fracture zones, fault development zones, large excavation bodies (such as goafs and chambers), and stress concentration areas of roadways and stopes. Also, the drilling depth was approximately 4 times of roadway span, ensuring that the stress measuring points were located in the original rock stress zone, that is, the determined stress state was not disturbed by mining activities. In addition, to study the variation of in-situ stress state with depth, the measuring points of each mine were performed at least at three depth levels. During the stress relief process, the borehole strain data and the temperature change value of the measuring point measured by each strain gauge in the hollow inclusion are continuously and automatically recorded by the data collector. After the stress relief is completed, the stress relief curve (Fig. 2) is obtained accordingly, which can be used to check the working state of each strain gauge. The information on rock elastic modulus and Poisson’s ratio is needed to convert the measured strain value into stress. Based on the OC test results and the indoor biaxial pressure test and temperature calibration results, the three-dimensional stress state in terms of the magnitude and direction in the host rock was calculated using the equations provided by Worotnicki and Walton^26^. In the calculation of in-situ stresses, a new double-iteration method with the developed stress inversion program was adopted to solve the *K* coefficients, elastic modulus, and Poisson’s ratio of rocks in the measuring points, which ensures the reliability and accuracy of the calculation results. The calculated stress tensors for the Shuichang iron mine and the Sanshandao gold mine are listed in Tables 1 and 2, respectively. To assess the quality of the determined OC stress data and ensure the comparability of OC and hydraulic fracturing stress measurements which will be described below, the OC stress indicators in the two mines were first examined by the internationally accepted World Stress Map (WSM) quality ranking system^27^ and were ranked into categories B and C.

**Table 1:** OC stress measurements in the Shuichang iron mine.

No	Depth (m)	Maximum principal stress *σ*_1_			Intermediate principal stress *σ*_2_			Minimum principal stress *σ*_3_		
		Value (MPa)	Direction (°)	Dip (°)	Value (MPa)	Direction (°)	Dip (°)	Value (MPa)	Direction (°)	Dip (°)
1	81	4.07	272.2	− 7.3	2.38	3.9	− 13.3	2.16	154.2	− 74.8
2	91.5	4.26	90.6	− 0.8	2.86	180.6	− 2.9	2.68	344.9	− 87.0
3	56	3.68	98.9	− 7.2	2.33	189.7	− 6.2	2.03	319.8	80.5

**Table 2:** OC stress measurements in the Sanshandao gold mine.

No	Depth (m)	Maximum principal stress *σ*_1_			Intermediate principal stress *σ*_2_			Minimum principal stress *σ*_3_		
		Value (MPa)	Direction (°)	Dip (°)	Value (MPa)	Direction (°)	Dip (°)	Value (MPa)	Direction (°)	Dip (°)
1	75	6.01	288.50	− 6.30	3.81	198.00	− 4.90	2.56	250.40	82.00
2	150	7.73	280.90	− 5.20	5.48	9.40	16.60	4.50	27.70	72.50
3	420	19.27	284.10	− 21.30	11.05	18.50	− 11.10	10.88	134.40	− 65.70
4	420	19.39	120.40	− 14.90	10.92	169.20	68.10	9.44	34.70	15.80
5	510	24.55	129.00	4.00	16.35	− 138.00	2.00	14.49	133.00	− 85.00
6	510	24.64	− 111.00	3.00	15.68	155.00	82.00	15.02	161.00	− 10.00
7	555	25.71	− 45.00	− 13.00	14.00	14.00	73.00	13.00	50.00	− 20.00
8	600	28.88	103.00	1.00	16.54	10.00	76.00	14.77	13.00	− 8.00
9	600	30.17	110.00	− 16.00	18.83	24.00	− 11.00	16.94	236.00	− 70.00
10	645	29.57	112.00	− 3.00	19.56	− 177.00	− 80.00	15.48	− 156.00	− 9.00
11	690	31.50	− 80.00	2.00	19.08	230.00	− 79.00	17.54	10.00	− 10.00
12	690	29.77	− 83.00	4.00	20.84	− 8.00	− 74.00	19.63	8.00	15.00
13	750	33.22	119.00	− 10.00	19.93	− 89.00	− 82.00	17.10	208.00	− 8.00
14	750	32.78	105.60	− 0.59	19.6	18.70	79.20	16.68	15.50	− 10.80
15	780	30.72	133.30	− 14.90	26.41	− 135.60	− 4.17	18.09	149.7	74.50
16	795	48.93	164.09	3.00	23.15	74.41	− 5.97	21.66	47.22	83.29
17	825	46.95	40.06	3.84	28.88	− 49.55	− 5.77	26.49	− 83.43	83.06
18	960	41.63	145.30	− 10.30	26.79	165.50	60.30	25.42	200.20	− 6.21

### Hydraulic fracturing

The hydraulic fracturing (HF) technique was also employed to measure the in-situ stresses in the Shuichang iron mine and the Sanshandao gold mine. The HF, which originated in the 1940s, is an oilfield stimulation technology, aiming at increasing the production of low-permeability oil-bearing layers by pressurizing a part of the wellbore^28^. Hubbert and Willis^29^ developed the classic concept of the HF pressure record interpretation in 1957, and later some scholars, such as Scheidegger^30^ and Rummel and Jung^31^, presented the classic HF method. After decades of development, the HF theory and equipment have been further improved and is now a well-established technique for determining in-situ stresses at depth. The reported in-situ stress measurement depth using the HF technique reached 9066 m, which was located in the KTB ultra-deep hole^15^.

**Figure 3:**
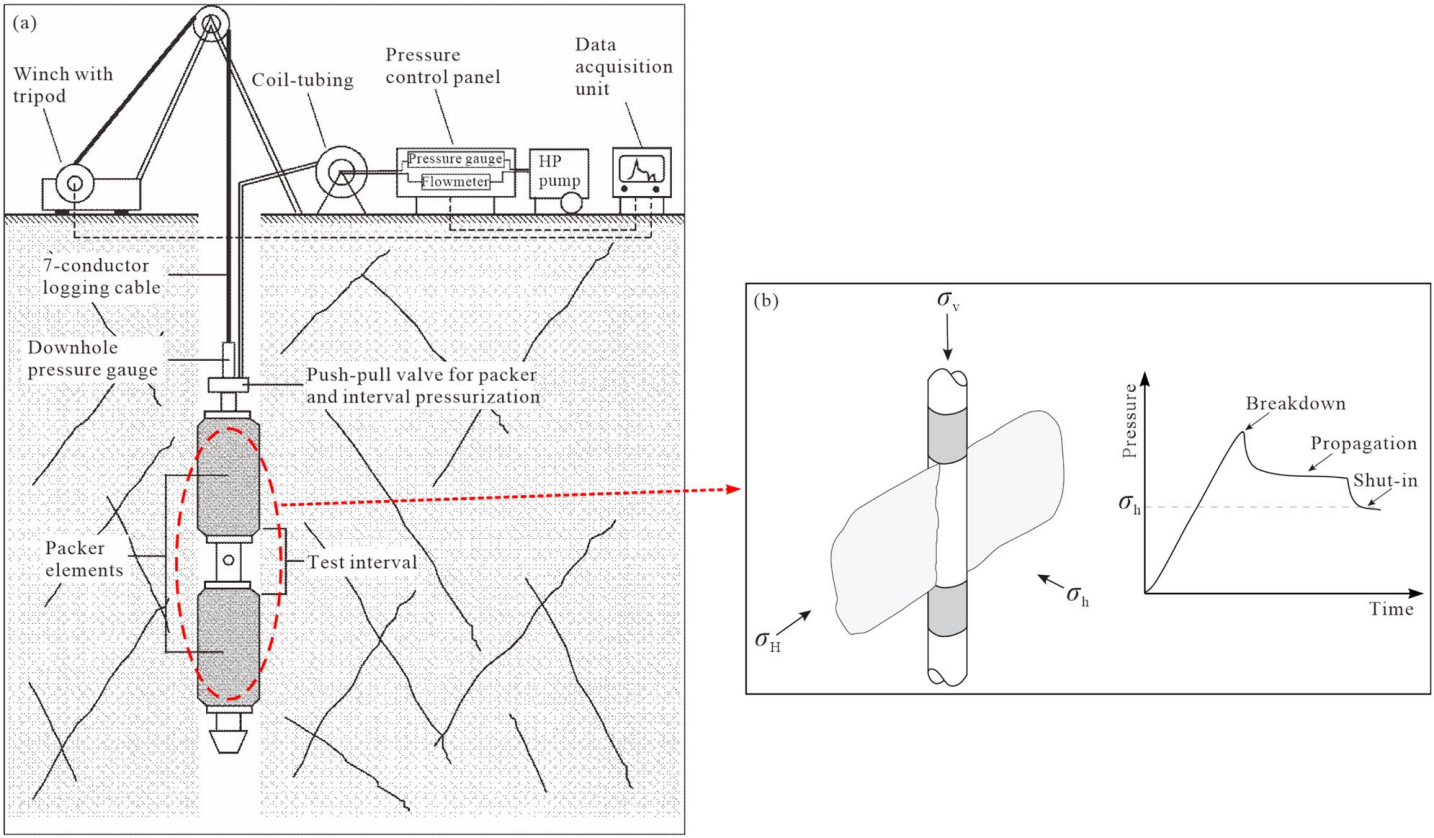
Schematic diagram of HF system (**a**) (after Ljunggren et al.^12^) and the HF crack propagation direction under the three-dimensional stress condition and the typical pressure–time record on the first injection/shut-in cycle (**b**) (after Lakirouhani et al. ^34^).

The HF method involves fixing a pair of expandable rubber packers at a predetermined drilling depth, isolating a small interval of the borehole as a measurement location, and then pumping high-pressure fluid into the testing interval to actively fracture the surrounding rocks, thereby inducing the formation of artificial tensile fractures at the borehole wall^32^, as illustrated in Fig. 3. The fracture plane is usually parallel to the borehole axis, and two fractures occur simultaneously at diametrically opposite positions around the borehole. The fracture will develop along the direction perpendicular to the minimum principal stress^17^, and the orientation of fractures can be determined based on the traces of fractures on the borehole wall. Thus, in a vertical or sub-vertical borehole where one principal stress is assumed to be parallel to the borehole, the direction of the initial crack coincides with the direction of the maximum horizontal stress. According to the basic elastic relationship between recorded pressures and in-situ stresses^19,33^, the stress magnitude at the testing location can be calculated. The stress direction can be determined by identifying the induced hydrofrac planes through a packer and compass or geophysical methods such as stratigraphic microscanners or borehole television viewers.

The classical HF method is essentially a two-dimensional stress measurement method, which is most suitable for measurement in vertical or sub-vertical boreholes. In practice, the vertical principal stress deviates from the vertical direction more or less. The analysis shows that as long as the “vertical” principal stress deviates from the vertical direction by no more than 25°, it is feasible to regard the principal stress as vertical and estimate it using the weight of overlying strata, and the resulting error is no more than 10%. In some special projects or special stages, such as deep open pit mines and the preliminary exploration stage of underground mines, where there are no underground facilities to approach the measuring points^12,35^, the application of the HF method to preliminarily measure the stress state in the engineering area and provide a basis for engineering design is the best choice. Unlike the OC method, the HF method does not use precision instruments to measure the strain at a certain point in the test borehole, but directly determines the stresses without relying on accurate values of elastic rock parameters, and theoretically, the depth is not limited by the length of the borehole. Moreover, the HF can estimate the stress in a large area and yield better average values than point measurements^33^. As a result, the HF technique has been widely used in continental scientific deep drilling, geodynamics research of active fault zones, and major rock mass engineering construction.

**Figure 4:**
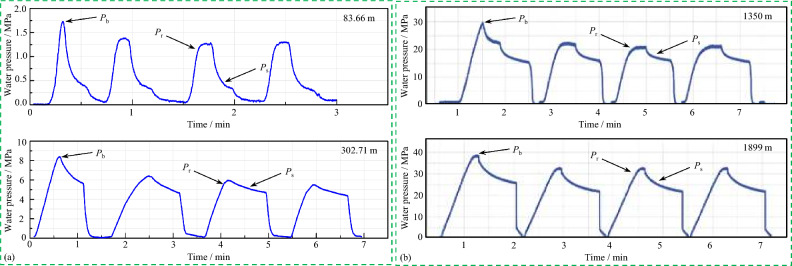
Typical pressure–time curves during the HF test in the Shuichang iron mine (a) and the Sanshandao gold mine (b) (after Zhu et al.^37^).

Different types of single-loop HF devices were applied to these two mines separately for stress measurement, following the ISRM suggested method^17^. HF measurements were successfully performed in three boreholes with effective four, three, and two fracturing sections, respectively, in the Shuichang iron mine, and the azimuths of the induced hydro-fractures in four test intervals were identified using an oriented impression packer. Moreover, 23 HF test intervals were satisfactorily tested in a deep borehole in the Sanshandao gold mine, and directional experiments were conducted at three test intervals through an oriented impression packer. During the tests, the relationship between injection pressure and time was measured synchronously. The shape of the pressure–time curve of each test interval in the two mines was standard and normative, and the pressure parameter points were clear (Fig. 4). The stress measurement accuracy highly depends on the obtained pressure–time records. Each test interval typically requires 3–5 cycles of opening and closing hydraulic fractures to more accurately determine borehole pressures and subsequent stress magnitudes. Based on the recorded original data, the stress-related hydraulic pressure parameters (i.e., breakdown pressure *P*_b_, shut-in pressure *P*_s_, and reopening pressure *P*_r_), tensile strength (*T*) of the rocks, and the corresponding stress magnitudes and directions in the two mines were derived and presented in Table 3 and Table 4, respectively. The relevant calculation or determination methods can be found in the introduction by Li et al.^19,36^. According to the WSM quality ranking system^27^, the HF stress data quality in the Shuichang iron mine can be ranked into the B, C, and D categories and that in the Sanshandao gold mine can be ranked as category C, revealing effective and significant information on the contemporary stress field. Note that the HF stress data of the Shuichang iron mine show notable scatter pattern, which is likely attributed to the following two reasons: (1) structures such as faults, synclines, and anticlines are highly developed in this mining area, with structures in various directions and periods crisscrossing, especially the multiple reactivations of faults and the widespread occurrence of joint and composite phenomena between faults, resulting in the changes in secondary and local stresses; and (2) the HF stress measuring point is shallow, within 303 m, which is easily influenced by terrain effects and historical geological structures, resulting in uneven stress distribution.

**Table 3:** HF stress measurements in the Shuichang iron mine.

Borehole	No	Depth (m)	Pressure parameters (MPa)					Principal stresses (MPa)			*σ*_H_ direction
			*P*_b_	*P*_r_	*P*_s_	*P*_0_	*T*	*σ*_H_	*σ*_h_	*σ*_v_	
BH-1	1	83.66	2.83	2.08	1.66	0.74	0.76	2.16	1.66	2.21	/
	2	116.37	5.93	4.50	4.08	1.06	1.43	6.68	4.08	3.07	N77.0°E
	3	155.68	12.57	7.57	6.98	1.44	5.00	11.93	6.98	4.11	N88.0°E
	4	181.83	12.91	9.13	7.83	1.70	3.78	12.65	7.83	4.80	/
BH-2	1	265.48	9.11	6.59	5.96	2.01	2.52	9.28	5.96	7.02	/
	2	274.81	11.72	6.89	6.26	2.10	4.83	9.79	6.26	7.26	N77.0°E
	3	302.71	11.78	8.42	8.00	2.38	3.36	13.21	8.00	8.00	/
BH-3	1	119.17	9.16	5.36	3.96	0.46	3.80	6.07	3.96	3.15	N77.0°E
	2	186.20	13.02	5.62	5.42	1.12	7.40	9.53	5.42	4.92	/

**Table 4:** HF stress measurements in the Sanshandao gold mine^37^.

No	Depth (m)	Pressure parameters (MPa)					Principal stresses (MPa)			*σ*_H_ direction
		*P*_b_	*P*_r_	*P*_s_	*P*_0_	*T*	*σ*_H_	*σ*_h_	*σ*_v_	
1	357.76	20.02	15.54	11.73	3.51	4.48	23.16	15.24	9.47	–
2	431.09	24.57	15.78	11.85	4.22	8.79	23.99	16.08	11.41	–
3	509.35	25.15	20.29	13.44	4.99	4.86	25.02	18.43	13.48	N55.5°W
4	608.26	25.16	18.82	14.35	5.96	6.34	30.20	20.31	16.39	–
5	665.33	24.34	21.39	14.75	6.52	2.95	29.37	21.27	17.60	–
6	881.70	30.22	23.09	16.29	8.64	7.14	34.42	24.93	23.33	–
7	957.10	25.86	22.54	16.57	9.38	3.33	36.56	25.95	25.32	–
8	1010.50	23.06	19.19	16.48	9.90	3.87	40.14	26.38	27.23	–
9	1097.50	30.43	25.72	20.19	10.76	4.71	45.62	30.95	29.04	N60.4°W
10	1166.41	34.84	25.72	20.50	11.43	8.90	46.99	31.93	30.86	–
11	1220.40	34.40	25.31	20.44	11.96	9.09	47.96	32.40	32.29	–
12	1275.80	32.79	23.63	19.52	12.50	9.16	47.43	32.02	34.38	–
13	1350.00	29.30	21.84	18.62	13.23	7.47	47.25	31.85	35.72	–
14	1408.00	28.02	23.36	19.32	13.80	4.66	48.40	33.12	37.26	–
15	1473.18	31.93	24.09	20.59	14.44	7.85	52.12	35.03	38.98	–
16	1512.50	31.01	24.98	20.75	14.82	6.04	52.11	35.58	40.02	N58.4°W
17	1594.60	31.82	26.59	22.12	15.63	5.23	55.40	37.75	42.19	–
18	1643.63	38.98	29.43	24.70	16.11	9.55	60.79	40.81	44.30	–
19	1689.50	37.83	28.31	24.46	16.56	9.52	61.63	41.02	44.70	–
20	1756.80	34.89	30.65	26.39	17.22	4.24	65.72	43.60	46.48	–
21	1792.70	32.69	27.70	24.83	17.57	4.99	64.37	42.40	47.43	–
22	1839.00	37.35	28.73	25.43	18.02	8.62	65.58	43.45	49.56	–
23	1899.00	42.47	33.10	28.45	18.61	9.37	70.86	47.06	50.25	–

## Comparison and evaluation of OC and HF stress measurements

The in-situ stress measurements were conducted using two different techniques in the same area, so the measurement results obtained through these two methods are comparable, as the geological tectonic background is considered to be the same. Moreover, the stress measurement results determined in these two mines are very comparable with other stress indicators or measurement results in their respective nearby areas^38–44^, which proves the effectiveness and applicability of the stress data in this study. The comparison and evaluation of the various stress measurements afford a valuable opportunity to evaluate the performance of measurement techniques and the reliability of measurement results.

### Stress magnitude

The magnitude and direction of the three principal stresses obtained by the OC method in these two mines were actually measured and calculated without any prior assumptions. In the OC data (Tables 1 and 2), there are indeed two principal stresses located in the horizontal or sub-horizontal direction at each measuring point and their included angles with the horizontal plane are basically less than 17°, which are regarded as the maximum (*σ*_H_) and minimum (*σ*_h_) horizontal principal stresses, respectively; the remaining principal stress is close to the vertical or sub-vertical direction, and the included angle with the horizontal plane almost exceeds 70°, which is deemed the vertical (*σ*_v_) principal stress. The dip angle of vertical principal stress measured by the OC method coincides well with the dip angle of the borehole during the HF measurement, which implies that the assumption that a principal stress component is vertical in the HF stress measurement is reasonable.

**Figure 5:**
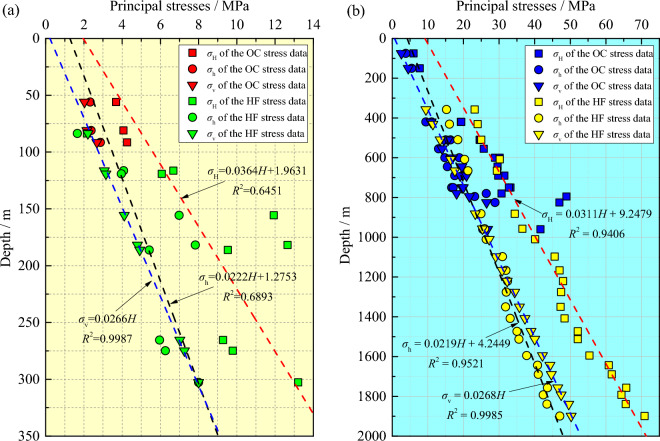
Stress–depth profiling in the Shuichang iron mine (**a**) and the Sanshandao gold mine (**b**).

One method commonly used for indirectly comparing stress results obtained by different measurement techniques is to use regression approaches to establish the distribution equations of principal stresses along fixed orientations based on the stress measurement results obtained by one measurement technique, and then check the degree of conformity between the stress measurement results obtained by another measurement technique and the equations. Both the OC and HF stress magnitude data derived from the two mines are synchronously plotted in Fig. 5 as a function of depth, and different variation forms of the three principal stresses with depth using the OC and HF data are listed in Table 5. In the Shuichang iron mine, horizontal stresses *σ*_H_ and *σ*_h_ of the OC + HF data exhibit a poor linear growth relationship with depth, and the correlation coefficients are correspondingly low. This indicates that the two kinds of data in this mine do not obey the same distribution model very well within the test depth range. Even, the linear relationship between independent OC and HF data and depth is worse, as shown in Table 5. In particular, the HF stress magnitudes vary greatly both laterally and vertically along the regression line. This may be attributed to the fact that the OC and HF stress data measured in this mine are less and cannot accurately reflect the distribution law of in-situ stress in the vertical direction. Nevertheless, the relationship between the horizontal stresses and depth is still positively correlated. Moreover, the vertical stress *σ*_v_ of the OC + HF data shows very little variation within the narrow depth ranges, with a correlation coefficient of 0.9987, implying that the measured real vertical stress is roughly equivalent to the gravitational stress calculated through the average bulk density.

**Table 5:** Regression equations between the principal stresses and depth and the number of stress regimes in the two mines.

Mine name	Regression equations			Stress regimes			
	OC data	HF data	OC + HF data	*σ*_H_ > *σ*_h_ > *σ*_v_	*σ*_H_ > *σ*_v_ > *σ*_h_	*σ*_v_ > *σ*_H_ > *σ*_h_	Data
Shuichang iron mine	*σ*_H_ = 0.0162*H* + 2.7691 (*R*^2^ = 0.9988)	*σ*_H_ = 0.0317*H* + 3.1020 (*R*^2^ = 0.4801)	*σ*_H_ = 0.0364*H* + 1.9631 (*R*^2^ = 0.6451)	3	0	0	OC
	*σ*_h_ = 0.0126*H* + 1.5651 (*R*^2^ = 0.6147)	*σ*_h_ = 0.0193*H* + 1.9630 (*R*^2^ = 0.5338)	*σ*_h_ = 0.0222*H* + 1.2753 (*R*^2^ = 0.6893)	6	3	0	HF
	*σ*_v_ = 0.0295*H* (*R*^2^ = 0.9877)	*σ*_v_ = 0.0264*H* (*R*^2^ = 1)	*σ*_v_ = 0.0266*H* (*R*^2^ = 0.9987)	9	3	0	OC + HF
Sanshandao gold mine	*σ*_H_ = 0.0461*H* + 0.9467 (*R*^2^ = 0.8710)	*σ*_H_ = 0.0299*H* + 10.1420 (*R*^2^ = 0.9765)	*σ*_H_ = 0.0311*H* + 9.2479 (*R*^2^ = 0.9406)	8	10	0	OC
	*σ*_h_ = 0.0271*H* + 0.4103 (*R*^2^ = 0.8317)	*σ*_h_ = 0.0193*H* + 7.9863 (*R*^2^ = 0.9824)	*σ*_h_ = 0.0219*H* + 4.2449 (*R*^2^ = 0.9521)	10	13	0	HF
	*σ*_v_ = 0.0278*H* (*R*^2^ = 0.9930)	*σ*_v_ = 0.0266*H* (*R*^2^ = 0.9999)	*σ*_v_ = 0.0268*H* (*R*^2^ = 0.9985)	18	23	0	OC + HF

In the Sanshandao gold mine, both the horizontal and vertical stresses of the OC + HF data exhibit a good linear growth relationship with depth (Fig. 5), and the correlation coefficients reach 0.9406, 0.9521, and 0.9985, respectively, which indicates a remarkable similarity in principal stress magnitudes between the OC and HF data in this mine. As such, they strictly follow the same distribution equation, and the reliability of the measurement is satisfactory. Furthermore, the three principal stresses of the independent OC and HF data also show very little scatter and increase linearly with depth, and the measured vertical stress from the OC data is in good agreement with the predicted one from the HF data in this mine (Table 5). Hence, there are only minor differences between the OC and HF data with respect to the principal stress magnitude distribution with depth.

On the other hand, for the OC and HF stress data in the two mines, the difference between *σ*_H_ and *σ*_v_ increases with increasing depth, which reflects that *σ*_H_ dominates the modern stress field, and further supports the view that the formation and evolution of stress field are controlled by horizontal tectonic movement. Also, both types of horizontal stress data from the two mines show that the surface stress level is not zero, which is a pathological phenomenon that may be related to the erosion of crustal surface materials^36^. The high constant term and gradient amplitude in the derived linear regression equations show that the surface tectonic action is strong, and the horizontal tectonic stress increases sharply with the increase of depth.

Based on the *σ*_H_, *σ*_h_, and *σ*_v_ magnitudes, the faulting stress regime in the Shuichang iron mine is primarily characterized by *σ*_H_ > *σ*_h_ > *σ*_v_ within the tested depths, as indicated by the OC and HF stress data in this mine (Table 5). This stress regime favors the formation and motion of the thrust fault. Moreover, two types of faulting stress regimes (*σ*_H_ > *σ*_h_ > *σ*_v_ and *σ*_H_ > *σ*_v_ > *σ*_h_) are identified in the Sanshandao gold mine based on the OC and HF stress data, with the prevailing *σ*_H_ > *σ*_v_ > *σ*_h_ (Table 5). In this case, the formation and movement of the thrust and strike-slip faults are favorable. Note that no normal faulting stress regimes (*σ*_v_ > *σ*_h_ > *σ*_H_) are revealed by the OC and HF stress data in the two mines. It can be seen that in each mine, the independent OC and HF data reveal the same dominant faulting stress regime, which is generally identical to the motion features of the fault structure in each mine and in North China region^4,45^.

**Figure 6:**
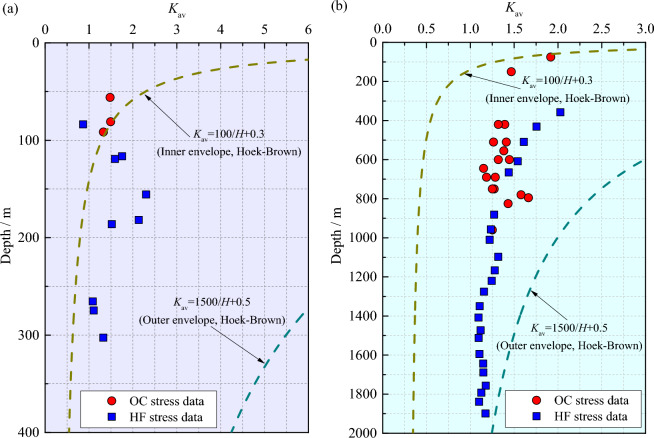
*K*_av_ values of the OC and HF stress data in the Shuichang iron mine (**a**) and the Sanshandao gold mine (**b**).

In addition, the ratio of average horizontal principal stresses to vertical stress (i.e., *K*_av_ = (*σ*_H_ + *σ*_h_)/2*σ*_v_) can not only reflect the relationship between the three principal stresses in the crust but also represent the compression degree of the lithosphere, and a higher *K*_av_ value corresponds to the high compression. *K*_av_ values of the OC and HF stress data in the two mines are calculated and plotted as functions of depth in Fig. 6. In the Shuichang iron mine, the nonlinear decrease in *K*_av_ with increasing depth is not significant, and the reason for this phenomenon may be due to the limited and scattered stress data. Nevertheless, *K*_av_ in this mine appears to approach 1.0 with increasing depth. Comparatively, *K*_av_ shows a more characteristic nonlinear downward trend with the increase of depth in the Sanshandao gold mine, approaching 1.0. Accordingly, it can be deduced that a quasi-hydrostatic pressure field may exist in the deeper crust. Moreover, the correlation between the *K*_av_ and depth has been revealed by Brown and Hoek ^46^ using the statistics of 116 sets of global stress databases, and the inner and outer envelopes of the correlation between the *K*_av_ and depth are confined in the form of hyperbolas, as embedded in Fig. 6. Evidently, the *K*_av_ values yielded from the Shuichang iron mine lie in the outer envelope but exceed the inner envelope when the depth less than 100 m, but the difference is small. This may be because the in-situ stress measurement at a depth of less than 100 m is easily related to nontectonic processes such as topography ^3,47^, resulting in somewhat stress anomalies. Furthermore, the *K*_av_ values derived from the Sanshandao gold mine lie in the inner and outer envelopes but are slightly larger/lower than those indicated by the inner/outer envelopes, showing a similar evolutionary trend.

It is worth emphasizing that the HF stress measurements show more scatter at a given depth in the Shuichang iron mine than the OC stress measurements. In the Sanshandao gold mine, however, it is the reverse trend where the OC stress measurements present scatter in the data while the HF stress measurements are remarkably consistent. This phenomenon may be because the rock mass exposed in the OC measurement in the Shuichang iron mine is relatively complete, and the spatial horizontal distance between the three measuring points is only 50.5–81.8 m, so the stress state revealed is relatively uniform. However, in the HF test, the exposed deeper rock mass is somewhat fragmented due to the widespread distribution of faults, synclines, and anticlines in the mining area, and the stress was tested in three different boreholes, which may lead to the scattering of stress measurements. In the Sanshandao gold mine, the OC measurements were performed at multiple depth levels and different locations within the mining area; the lithology of different measurement points also varies, and there may be local force sources causing variations in stress states. The HF test involves measuring test sections at different depth levels in the same deep borehole, and the integrity of the drilled rock cores is relatively high without visible cracks, which is beneficial for obtaining uniform stress. In fact, the above situation is normally expected from the field stress measurements across a broad mining area, which has also been observed in many other regions worldwide^48–50^.

### Stress orientation

**Figure 7:**
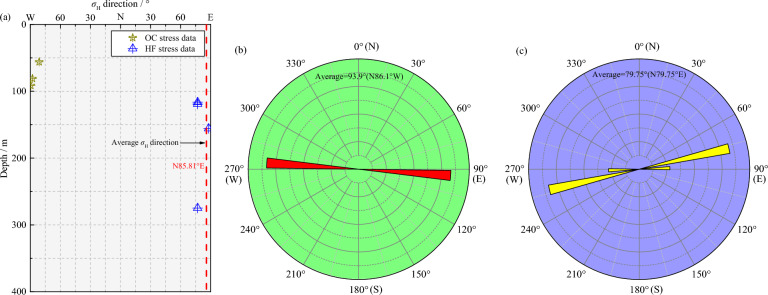
The maximum principal stress orientations determined in the Shuichang iron mine using the OC and HF techniques in a depth profile (**a**), a rose diagram for OC stress direction statistics (**b**), and a rose diagram for HF stress direction statistics (**c**).

**Figure 8:**
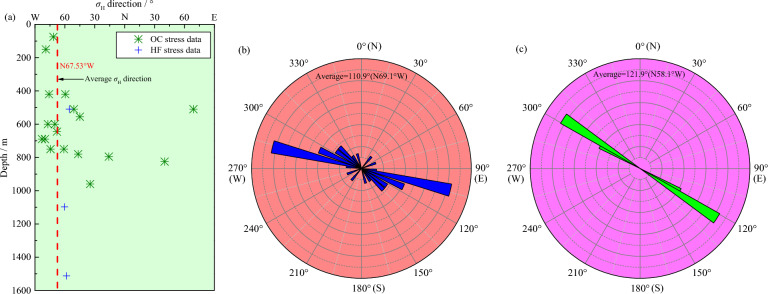
The maximum principal stress orientations obtained in the Sanshandao gold mine via the OC and HF methods in a depth profile (**a**), a rose diagram for OC stress direction statistics (**b**), and a rose diagram for HF stress direction statistics (**c**).

**Figure 9:**
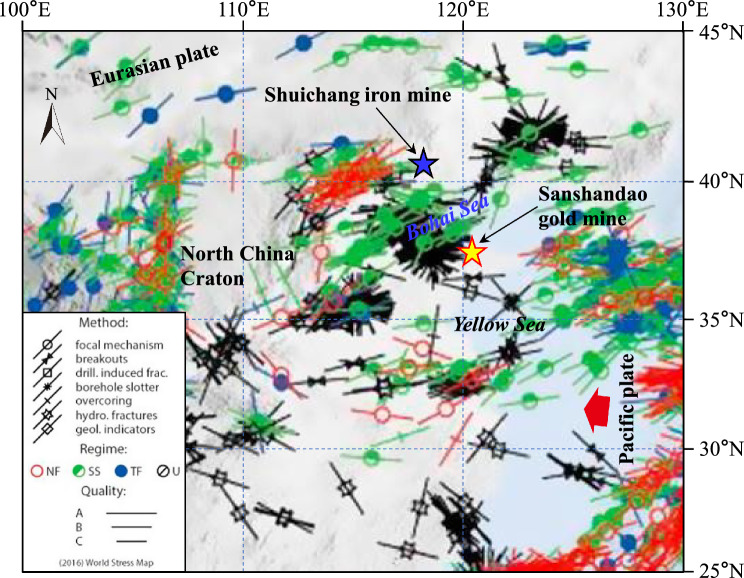
Modern tectonic stress field orientations interpreted by various stress indicators in and around the Shuichang iron mine and Sanshandao gold mine (after Hu et al.^58^).

The stress orientation is a crucial component of stress tensors and a useful evaluation index. The maximum principal stress normally regulates the modern crustal stress environment in the shallow part ^36^. The maximum principal stress orientations measured in the two mines using the two techniques are visualized by rose diagrams, as illustrated in Fig. 7 and Fig. 8, respectively. In the Shuichang iron mine, *σ*_H_ orientations determined by the OC and HF methods are mainly within the NEE–SWW or nearly E–W direction, which changes little with the depth (Fig. 7a). Specifically, the maximum principal stress orientation derived from the OC method covers a narrow range of azimuths and is dominantly oriented in the N81.1°W–N89.4°W direction, averaging N86.1°W, with a standard deviation of 4.40° (Fig. 7b). Moreover, the maximum principal stress direction yielded from the HF method is reasonably well constrained and is predominantly in the N77.0°E–N88.0°E direction, averaging N79.75°E, with a standard deviation of 5.50° (Fig. 7c). Evidently, the included angle of the average stress orientation obtained by the two methods is only 14.15°, indicating a dominant stress orientation between the NWW–SEE and NEE–SWW or nearly E–W direction, which is identical to the prevailing regional tectonic stress field direction indicated by the other stress indicators (Fig. 9), such as neotectonic movement and modern focal mechanism solutions^51–55^. Hence, the maximum principal stress orientations in the Shuichang iron mine obtained by the two different methods are comparatively similar.

In the Sanshandao gold mine, *σ*_H_ orientations determined by the OC and HF methods are mainly within the NWW–SEE direction, the OC stress direction data shows a certain degree of fluctuation with increasing depth, while the HF stress direction data seems to be independent of depth (Fig. 8a). Specifically, the maximum principal stress orientation provided by the OC technique is largely constrained between N30°W and N90°W, averaging N69.1°W, with a standard deviation of 27.56° (Fig. 8b). The large-scale deflection of the *σ*_H_ direction with depth in the mine indicates that the *σ*_H_ direction is significantly affected by local or regional geological factors and stress sources because the mine is located in the land-sea boundary area with multiple large active faults distributed around it ^19^, which leads to a rotation or decoupling of the predominant NWW–SEE-trending regional tectonic stress mode and then the occurrence of local variations in the stress direction. This also implies that there may be localized control on the *σ*_H_ direction in this mine. In addition, the maximum principal stress orientation interpreted by the HF technique is highly concentrated in a small range, ranging from N55.5°W to N60.4°W, averaging N58.1°W, with a standard deviation of 2.46° (Fig. 8c), which only differs by 11° from the average stress direction calculated by the OC technique, showing a good consistency. Remarkably, these two stress indicators all represent the present-day stress field of the mine appears to be primarily dependent on the NWW–SEE-oriented regional tectonic stress field interpreted from near-field and far-field stress indicators (Fig. 9), such as structural trace analysis, faulting pattern, and focal mechanism solutions ^32,38,55–57^. Thus, the stress measurements indicated by the OC and HF methods agree fairly well, despite certain scatter in the stress data, which represents regional stress conditions.

In addition, the stress directions identified by these two stress measurement methods all demonstrate that the tectonic stress fields of these two mines are dominated by horizontal tectonic stress, which are both compressive stresses. Moreover, within the same depth or approximate depth of the horizontal plane of the same mine, there are certain changes in the magnitude and direction of the in-situ stress obtained by the two stress measurement methods at different positions; however, overall, they are still relatively similar and there is no sudden change phenomenon, indicating that the stress field of each mine is still relatively uniform.

In summary, the stress directions obtained using different measurement methods in these two mines are considerably similar, and it can be confidently believed that the stress measurements are credible. The high consistency of the stress directions in the two mines is not an accidental coincidence, which, on the one hand, means that each mine is controlled by the identical regional tectonic stress field with the same type of stress pattern under the same geological environment, and on the other hand, it implies that the performance of these two measurement techniques is quite reliable.

## Bayesian quantification of OC and HF stress measurements

### Improved Bayesian regression approach

Although OC and HF stress measurements are conducted in the same mine, they cannot be carried out at the same point, so strictly speaking, it is impossible to directly compare and evaluate their measurement results. Furthermore, stress measurement results are usually influenced by many factors, and most of them are variable. Due to the limitations of mining engineering conditions, the selection and layout of measurement points have significant limitations. In addition, the geological conditions of the mining areas are quite complex, and the stress state at each point has strong randomness. Consequently, it is not perfect to compare the distribution characteristics of stress magnitude and direction determined by different measurement techniques by using traditional statistical approaches based on the measured data of only a few points.

Because the position and depth of in-situ stress measured by OC and HF methods in the two mines are different, to verify whether the stress data in each mine measured by these two methods follow the same distribution, it is necessary to align the two types of data first. Considering some uncertainties such as inevitable errors that may occur in in-situ stress measurement, an improved Bayesian linear regression approach is proposed for modeling and data alignment in this study. This approach is more flexible and comprehensive in statistical modeling and provides more stable estimates in dealing with small sample problems by introducing prior information^59^.

For a given data set $D = \left\{ {X,Y} \right\}$, where $X = \left( {x_{1} ,x_{2} , \cdots ,x_{N} } \right)^{T}$ and $Y = \left( {y_{1} ,y_{2} , \cdots ,y_{N} } \right)^{T}$, the basic model of linear regression can be expressed as

1 $$y = f\left( x \right) + \varepsilon = \omega^{T} x + \varepsilon$$

where $$\varepsilon \sim N\left( {0,\sigma^{2} } \right)$$.

In Bayesian linear regression, *ω* and *y* are regarded as unknown random variables, and then inference and prediction are made step by step. In the inference stage, the distribution of parameter *ω* is derived based on Bayesian formulas, while in the prediction stage, the target distribution *y* is predicted based on the inferred distribution of parameter *ω*.

In the inference stage, according to the Bayesian formula, it can be concluded that:

2 $$P\left( {\omega |X,Y} \right) = \frac{{P\left( {\omega ,Y|X} \right)}}{{P\left( {Y|X} \right)}} = \frac{{P\left( {Y|\omega ,X} \right)P\left( \omega \right)}}{{\int {P\left( {Y|\omega ,X} \right)P\left( \omega \right)d\omega } }}$$

According to Eq. (2), it can be obtained that:

3 $$P\left( {\omega |X,Y} \right) \propto P\left( {Y|\omega ,X} \right)P\left( \omega \right)$$

in which, the likelihood part is:

4 $$P\left( {Y|\omega ,X} \right) = \prod\limits_{i = 1}^{N} {P\left( {y_{i} |\omega ,x_{i} } \right)} = \prod\limits_{i = 1}^{N} {\frac{1}{{\left( {2\pi } \right)^{0.5} \sigma }}} \exp \left\{ { - \frac{1}{{2\sigma^{2} }}\left( {y_{i} - \omega^{T} x_{i} } \right)^{2} } \right\} = N\left( {X\omega ,\sigma^{ - 2} I} \right)$$

The prior part is assumed to be $P\left( \omega \right) = N\left( {0,\sum_{p} } \right)$, and the posterior distribution $P\left( {\omega |X,Y} \right) = N\left( {\mu_{\omega } ,\sum_{\omega } } \right)$ can be deduced according to the self-conjugation of Gaussian distribution. After substitution, $\mu_{\omega } = \sigma^{ - 2} A^{ - 1} X^{T} Y$ and $\sum_{\omega } = A^{ - 1}$, where $A = \sigma^{ - 2} X^{T} X + \sum_{p}^{ - 1}$, can be obtained using the method of completing the square.

In the prediction stage, the linear regression model is:

5 $$y* = f\left( {x*} \right) + \varepsilon$$

According to the properties of Gaussian distribution, there are:

6 $$y^{ * } \sim N\left( {x^{ * T} \mu_{\omega } ,x^{ * T} \sum_{\omega } x^{ * } + \sigma^{2} I} \right)$$

After data completion, the probability density plot is used to judge whether the OC and HF stress data in the same mine follow the same distribution, thereby validating the consistency and effectiveness of the two types of stress data. The probability density function is a function that describes the probability distribution of random variables. For continuous random variables, the value of the probability density function at a certain point does not represent probability, but rather probability density. The probability density function must satisfy the conditions of nonnegativity and normalization.

For continuous random variable *E*, the probability density function is *p*(*e*), where $e \in \left( { - \infty , + \infty } \right)$. For any real number *e*, there is

7 $$P\left( {E \le e} \right) = \int_{ - \infty }^{e} {p\left( t \right)dt}$$

**Figure 10:**
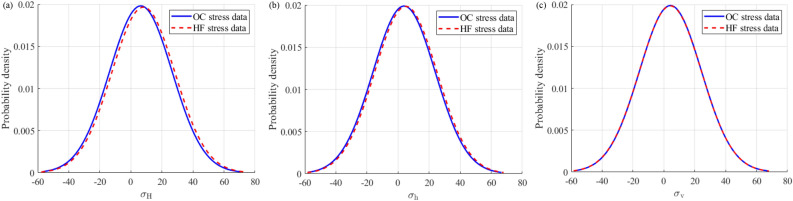
Probability density diagrams of the maximum (**a**), minimum (**b**), and vertical (**c**) principal stresses measured by the OC and HF methods in the Shuichang iron mine.

**Figure 11:**
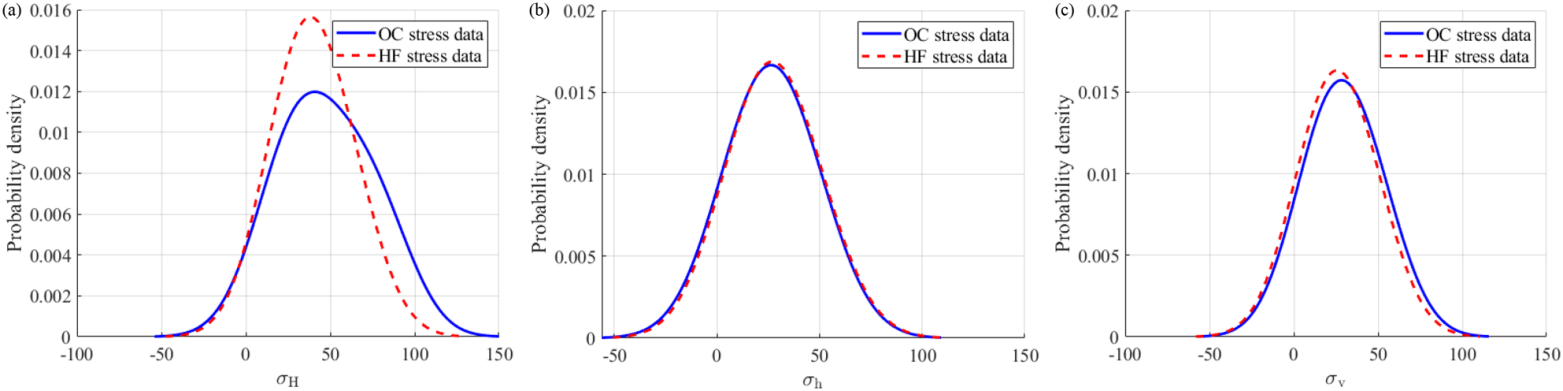
Probability density diagrams of the maximum (**a**), minimum (**b**), and vertical (**c**) principal stresses measured by the OC and HF methods in the Sanshandao gold mine.

The focus of this study is on the distribution of two types of stress data rather than prediction, so the prediction accuracy of the established Bayesian linear regression model does not require sufficient accuracy. Firstly, model the OC stress data of a mine and predict the depth information based on the HF stress data; at the same time, model the HF stress data in the mine and predict the depth information in the OC stress data. After completing the above steps, data alignment is achieved. Then, probability density plots are drawn for the stress data measured by the OC and HF methods in the two mines based on the true and predicted values corresponding to the depth information in the OC method as well as the true and predicted values corresponding to the depth information in the HF method, respectively, as shown in Figs. 10 and 11. It can be observed that the shapes of the probability density diagrams of the maximum, minimum, and vertical principal stresses measured by the OC and HF methods in the same mine are quite similar, and the expected values of the data are basically the same, that is, the curves almost overlap. Thus, it can be deduced that the stress data measured by the OC and HF methods in the same mine approximately follow the same distribution law. Note that the negative values of the principal stresses on the abscissa in Figs. 10 and 11 only indicate the probability of occurrence in the interval of (-∞, + ∞) and do not represent the real stress value. In addition, the result that the stress data measured by the OC and HF methods in the same mine basically follow the same distribution law determined by the probability density map is not contradictory to the relatively discrete distribution of the principal stresses with depth mentioned earlier because, in the Bayesian quantitative analysis, the depth data of the OC and HF methods are aligned first to estimate whether the distribution of stress obtained by these two methods at the same depth is the same.

### Distribution model validation

**Figure 12:**
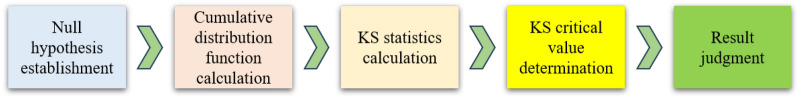
The inspection process of the improved KS statistical test for the distribution of OC and HF stress data.

To ensure the correctness of the probability density plots obtained by using the established Bayesian regression model, the distribution of OC and HF stress data is further verified through an improved Kolmogorov–Smirnov (KS) statistical test. KS test is a nonparametric statistical test method aimed at testing continuous distributions, which can usually be applied to compare the similarity of cumulative distributions between two datasets. The KS test converts the maximum difference between the two types of data distribution into a p-value and then compares the p-value with the selected significance level. If the p-value is less than the selected significance level, the null hypothesis is rejected and it is considered that these two types of data do not obey the same distribution; and vice versa. The inspection process adopted is as shown in Fig. 12, specifically as follows:

1. Establish the null hypothesis, which assumes that two types of data come from the same distribution.
2. For each group of data, calculate the cumulative distribution function value of each data point. For the sorted data points, the cumulative percentage of each point is calculated.
3. Calculate the KS statistic $D = \max \left| {F_{1} \left( x \right) - F_{2} \left( x \right)} \right|$, which is the maximum vertical distance between two sets of cumulative distribution functions, where $F_{1} \left( x \right)$ and $F_{2} \left( x \right)$ are the cumulative distribution functions of the two sets of data, respectively.
4. According to the selected significance level (chosen as 0.01 in this study), find the corresponding KS critical value.
5. Judging the result, that is, if the calculated KS statistic (*D*) is greater than the critical value, reject the null hypothesis and assume that the two sets of data do not come from the same distribution. Conversely, if the calculated KS statistic (*D*) is less than or equal to the critical value, the null hypothesis is accepted and the two groups of data are considered to come from the same distribution.

**Figure 13:**
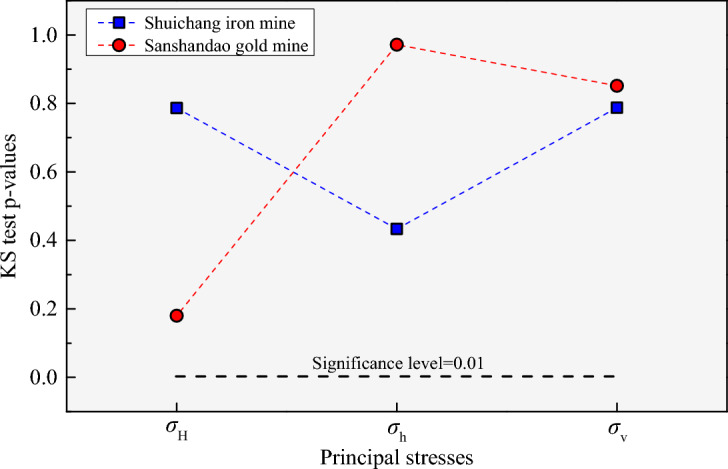
Comparison between the KS test p-values for the three principal stresses of the two mines and the selected significance level.

The supplemented data is subjected to a KS test using a self-designed MATLAB program. The results indicate that the OC and HF stress data accept the null hypothesis; in other words, the OC and HF stress data follow the same distribution. Moreover, the KS test p-values for the maximum, minimum, and vertical principal stresses of the Shuichang iron mine are 0.7864, 0.4333, and 0.7876, respectively, and correspondingly, the KS test p-values for the three principal stresses of the Sanshandao gold mine are 0.1800, 0.9713, and 0.8514, respectively (Fig. 13). All the obtained p-values are larger than the selected significance level of 0.01. Through intuitive analysis of probability density plots and KS test theory, it is believed that the OC and HF stress data in the same mine follow the same distribution law. This implies that the in-situ stress measurement results obtained by OC and HF methods in these two mines have good consistency. Hence, the performance of the two stress measurement techniques and the reliability of the measured data are satisfactory.

It should be emphasized that the number of stress measurement points in the two mines is relatively small and the depth coverage is not sufficient (especially for OC stress measurements), which may affect the representativeness and accuracy of stress data and distribution model. Moreover, the Bayesian quantification approach and Kolmogorov–Smirnov test are only used to compare the stress data measured in these two mines, and the universality of the results needs to be further supplemented. However, the proposed method in this study provides an effective means for comparing the data obtained by different stress measurement techniques and can provide an evaluation of the performance and reliability of different stress measurement techniques. Also, our findings can provide recommendations or suggestions for future research or applications. In the future, the Bayesian quantification approach and the inspection method will be used to compare and analyze more stress data covering a larger depth range in more engineering areas to continuously revise and optimize the approach.

## Conclusions

1. There are two principal stresses of the OC data located in the horizontal or sub-horizontal direction at each measuring point, and the remaining principal stress is close to the vertical or sub-vertical direction. The dip angle of vertical principal stress measured by the OC method coincides well with the dip angle of the borehole during the HF measurement, which suggests that the assumption that a principal stress component is vertical in the HF stress measurement is reasonable.
2. The horizontal stresses of the OC + HF data in the Shuichang iron mine exhibit a poor linear growth relationship with depth, indicating that the two kinds of data in this mine do not obey the same distribution model very well within the test depth range. Both the horizontal and vertical stresses of the OC + HF data in the Sanshandao gold mine exhibit a good linear growth relationship with depth, which indicates a remarkable similarity in principal stress magnitudes between the OC and HF data in this mine. For the OC and HF stress data in the two mines, the difference between *σ*_H_ and *σ*_v_ increases with increasing depth, which reflects that *σ*_H_ dominates the modern stress field. The independent OC and HF data in the two mines reveal the same dominant faulting stress regime, which is generally identical to the motion features of the fault structure in each mine.
3. The *σ*_H_ orientation derived from the OC method in the Shuichang iron mine is dominantly oriented in the N81.1°W–N89.4°W direction, averaging N86.1°W, and the *σ*_H_ direction yielded from the HF method is predominantly in the N77.0°E–N88.0°E direction, averaging N79.75°E. The *σ*_H_ orientation provided by the OC technique in the Sanshandao gold mine is largely constrained between N30°W and N90°W, averaging N69.1°W, and the *σ*_H_ orientation interpreted by the HF technique ranges from N55.5°W to N60.4°W, averaging N58.1°W. Hence, the *σ*_H_ orientations in the two mines obtained by the two different methods are comparatively similar.
4. The shapes of the probability density diagrams of the maximum, minimum, and vertical principal stresses measured by the OC and HF methods in the same mine are quite similar, and the expected values of the data are basically the same, that is, the curves almost overlap. Moreover, all the obtained KS test p-values are larger than the selected significance level of 0.01. Thus, it can be deduced that the stress data measured by the OC and HF methods in the same mine approximately follow the same distribution law. The performance of the two stress measurement techniques and the reliability of the measured data are satisfactory.

## Data Availability

The datasets used during the current study are available from the corresponding author upon reasonable request.
